# 4-(4-Ethoxy­benz­yl)-1,3-oxazolidin-2-one

**DOI:** 10.1107/S160053680900957X

**Published:** 2009-03-19

**Authors:** Hong-Yong Wang, Min-Hao Xie, Shi-Neng Luo, Yong-Jun He, Pei Zou

**Affiliations:** a Jiangsu Institute of Nuclear Medicine, Wuxi 214063, People’s Republic of China

## Abstract

In the title compound, C_12_H_15_NO_3_, the ethoxy­benzyl ring plane forms a dihedral angle of 60.3 (4)° with the mean plane of the oxazolidine ring. The mol­ecules are linked through N—H⋯O hydrogen bonds into a chain running in the *b* direction.

## Related literature

For background literature, see: Chrzanowska & Rozwadowska (2004 ▶); Rozwadowska (1994 ▶); Scott & Williams (2002 ▶); Tussetschläger *et al.* (2007 ▶).
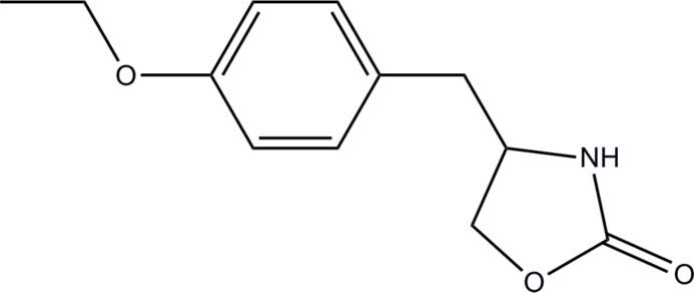


## Experimental

### 

#### Crystal data

C_12_H_15_NO_3_
*M* *_r_* = 221.25
Orthorhombic, 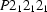

*a* = 5.7960 (12) Å
*b* = 9.924 (2) Å
*c* = 20.209 (4) Å
*V* = 1162.4 (4) Å^3^
*Z* = 4
Mo *K*α radiation
μ = 0.09 mm^−1^
*T* = 293 K
0.30 × 0.20 × 0.10 mm

#### Data collection

Enraf–Nonius CAD-4 diffractometer
Absorption correction: ψ scan (*CAD-4 Software*; Enraf–Nonius, 1989 ▶) *T*_min_ = 0.973, *T*_max_ = 0.991
2427 measured reflections
1246 independent reflections
904 reflections with *I* > 2σ(*I*)
*R*_int_ = 0.043
3 standard reflections every 200 reflections intensity decay: 1%

#### Refinement

*R*[*F* ^2^ > 2σ(*F* ^2^)] = 0.043
*wR*(*F* ^2^) = 0.150
*S* = 1.01
1246 reflections
146 parameters
H-atom parameters constrained
Δρ_max_ = 0.17 e Å^−3^
Δρ_min_ = −0.19 e Å^−3^

### 

Data collection: *CAD-4 Software* (Enraf–Nonius, 1989 ▶); cell refinement: *CAD-4 Software*; data reduction: *XCAD4* (Harms & Wocadlo, 1995 ▶); program(s) used to solve structure: *SHELXS97* (Sheldrick, 2008 ▶); program(s) used to refine structure: *SHELXL97* (Sheldrick, 2008 ▶); molecular graphics: *SHELXTL* (Sheldrick, 2008 ▶); software used to prepare material for publication: *SHELXL97*.

## Supplementary Material

Crystal structure: contains datablocks I, global. DOI: 10.1107/S160053680900957X/pv2144sup1.cif

Structure factors: contains datablocks I. DOI: 10.1107/S160053680900957X/pv2144Isup2.hkl

Additional supplementary materials:  crystallographic information; 3D view; checkCIF report

## Figures and Tables

**Table 1:** Hydrogen-bond geometry (Å, °)

*D*—H⋯*A*	*D*—H	H⋯*A*	*D*⋯*A*	*D*—H⋯*A*
N—H0*A*⋯O3^i^	0.86	1.99	2.845 (4)	171

Symmetry code: (i) 
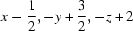
.

**Fig. 1.**
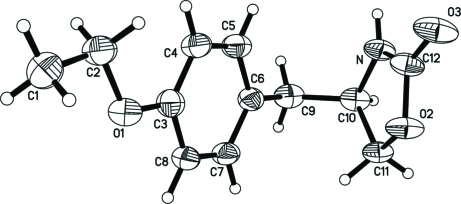
A view of the title compound with the atomic numbering scheme. Displacement ellipsoids were drawn at the 30% probability level.

## supplementary crystallographic information

### Comment

Tetrahydroisoquinoline alkaloids have received much interest because of their tremendous structural diversity and broad spectrum of biological and pharmaceutical activities (Rozwadowska, 1994; Scott & Williams, 2002; Chrzanowska & Rozwadowska, 2004). As part of our own work in this area, we prepared the title compound, (I), as an intermediate in the synthesis of tyrosine-derived *N*-[(phenylsulfonyl)alkyl]oxazolidinones as an extension of Petrini's methodology (Tussetschläger, *et al.*, 2007). The molecular structure of (I) is shown in Fig. 1. The dihedral angle between the mean planes of the C1 - C9/O1 ethoxybenzyl ring and the C10/N/C12/O2/C11/O3 oxazolidine ring is 60.3 (4)°. In the crystal structure, adjacent molecules are linked through N—H···O type hydrogen-bonding interactions resulting in chains running in the *b* direction (Table 1). The structure also contains non-classical hydrogen bonds of the type C—H···O linking the molecules into chains along the *a*-axis.

### Experimental

Sodium borohydride was added to a solution of 2-(*tert*-butoxycarbonylamino)-3-(4-ethoxyphenyl)propanoic acid (3.09 g, 10 mmol) in tetrahydrofuran (50 ml). Then methanol (5 ml) was slowly added to the resulting suspension and the temperature kept below 243 K. After the mixture was stirred for 1 h at room temperature, the excess reagent was destroyed by addition of acetic acid (1 ml). The solvent was evaporated, and the oily residue was diluted with water (50 ml) and extracted three times with ethyl acetate (25 ml) each. The combined organic extracts were wash with brine, dried with sodium sulfate, and concentrated *in vacuo*. The crude *tert*-butyl 1-(4-ethoxyphenyl)-3- hydroxypropan-2-ylcarbamate was obtained 2.7 g. Then *tert*-butyl 1- (4-ethoxyphenyl)-3-hydroxypropan-2-ylcarbamate (2.7 g) in THF (50 ml) was added to a suspension of sodium hydride (0.92 g, 23 mmol) in THF (120 ml) over a period of 20 min, stirred for 12 h, then quenched with a saturated solution of aqueous ammonium chloride (45 ml). The reaction mixture was then extracted three times with ethyl acetate (25 ml) each, the organic layers combined, washed with aqueous hydrochloric acid (60 ml, 5% solution), saturated NaHCO_3_ solution (60 ml), and brine (60 ml), and then dried over sodium sulfate. The solvent was then removed *in vacuo* to yield the title compound (1.81 g, 8.2 mmol) as a white solid. The title compound was crystalized by slow evaporation of a solution in methanol.

### Refinement

Positional parameters of all the H atoms bonded to C atoms were calculated geometrically and were allowed to ride on the C atoms to which they are bonded, with N—H = 0.86 and C—H = 0.93, 0.96, 0.97 and 0.98 Å for aryl, methyl, mehtylene and methine H atoms, respectively, and *U*_iso_(H) = 1.5*U*_eq_(methyl) and 1.2*U*_eq_(the rest) parent atoms. An absolute configuration could not be established by anomalous dispersion effects. Therefore, Fridel pairs (846) were merged.

### Crystal data

**Table:**

C_12_H_15_NO_3_	*F*(000) = 472
*M**_r_* = 221.25	*D*_x_ = 1.264 Mg m^−^^3^
Orthorhombic, *P*2_1_2_1_2_1_	Mo *K*α radiation, λ = 0.71073 Å
Hall symbol: P 2ac 2ab	Cell parameters from 25 reflections
*a* = 5.7960 (12) Å	θ = 9–12°
*b* = 9.924 (2) Å	µ = 0.09 mm^−^^1^
*c* = 20.209 (4) Å	*T* = 293 K
*V* = 1162.4 (4) Å^3^	Needle, colourless
*Z* = 4	0.30 × 0.20 × 0.10 mm

### Data collection

**Table:**

Enraf–Nonius CAD-4 diffractometer	904 reflections with *I* > 2σ(*I*)
Radiation source: fine-focus sealed tube	*R*_int_ = 0.043
graphite	θ_max_ = 25.3°, θ_min_ = 2.0°
ω/2θ scans	*h* = 0→6
Absorption correction: ψ scan (*CAD-4 Software*; Enraf–Nonius, 1989)	*k* = 0→11
*T*_min_ = 0.973, *T*_max_ = 0.991	*l* = −24→24
2427 measured reflections	3 standard reflections every 200 reflections
1246 independent reflections	intensity decay: 1%

### Refinement

**Table:**

Refinement on *F*^2^	Secondary atom site location: difference Fourier map
Least-squares matrix: full	Hydrogen site location: inferred from neighbouring sites
*R*[*F*^2^ > 2σ(*F*^2^)] = 0.043	H-atom parameters constrained
*wR*(*F*^2^) = 0.150	*w* = 1/[σ^2^(*F*_o_^2^) + (0.08*P*)^2^ + 0.28*P*] where *P* = (*F*_o_^2^ + 2*F*_c_^2^)/3
*S* = 1.01	(Δ/σ)_max_ < 0.001
1246 reflections	Δρ_max_ = 0.17 e Å^−^^3^
146 parameters	Δρ_min_ = −0.19 e Å^−^^3^
0 restraints	Extinction correction: *SHELXL97* (Sheldrick, 2008)
Primary atom site location: structure-invariant direct methods	Extinction coefficient: 0.031 (6)

### Special details

**Table:**

Geometry. All e.s.d.'s (except the e.s.d. in the dihedral angle between two l.s. planes) are estimated using the full covariance matrix. The cell e.s.d.'s are taken into account individually in the estimation of e.s.d.'s in distances, angles and torsion angles; correlations between e.s.d.'s in cell parameters are only used when they are defined by crystal symmetry. An approximate (isotropic) treatment of cell e.s.d.'s is used for estimating e.s.d.'s involving l.s. planes.
Refinement. Refinement of *F*^2^ against ALL reflections. The weighted *R*-factor *wR* and goodness of fit *S* are based on *F*^2^, conventional *R*-factors *R* are based on *F*, with *F* set to zero for negative *F*^2^. The threshold expression of *F*^2^ > σ(*F*^2^) is used only for calculating *R*-factors(gt) *etc*. and is not relevant to the choice of reflections for refinement. *R*-factors based on *F*^2^ are statistically about twice as large as those based on *F*, and *R*- factors based on ALL data will be even larger.

### Fractional atomic coordinates and isotropic or equivalent isotropic displacement parameters (Å^2^)

**Table:**

	*x*	*y*	*z*	*U*_iso_*/*U*_eq_	
N	−0.1179 (6)	0.5921 (3)	1.00790 (17)	0.0589 (9)	
H0A	−0.1581	0.6728	1.0182	0.071*	
O1	0.4026 (6)	0.3182 (3)	1.25901 (14)	0.0754 (9)	
O2	0.0990 (5)	0.4271 (3)	0.97230 (16)	0.0696 (9)	
O3	0.2494 (5)	0.6348 (3)	0.97241 (19)	0.0845 (11)	
C1	0.7072 (9)	0.3538 (5)	1.3348 (2)	0.0801 (14)	
H1A	0.7873	0.4207	1.3603	0.120*	
H1B	0.8134	0.3107	1.3051	0.120*	
H1C	0.6415	0.2878	1.3639	0.120*	
C2	0.5179 (9)	0.4202 (4)	1.2955 (2)	0.0725 (12)	
H2A	0.4105	0.4650	1.3250	0.087*	
H2B	0.5824	0.4869	1.2657	0.087*	
C3	0.2251 (8)	0.3587 (4)	1.21793 (19)	0.0596 (11)	
C4	0.1675 (9)	0.4911 (4)	1.2056 (2)	0.0651 (12)	
H4A	0.2504	0.5608	1.2252	0.078*	
C5	−0.0159 (8)	0.5190 (4)	1.1637 (2)	0.0643 (12)	
H5A	−0.0542	0.6086	1.1559	0.077*	
C6	−0.1449 (8)	0.4190 (4)	1.13291 (19)	0.0595 (11)	
C7	−0.0758 (10)	0.2863 (4)	1.1469 (2)	0.0648 (13)	
H7A	−0.1548	0.2156	1.1270	0.078*	
C8	0.1011 (10)	0.2573 (4)	1.1883 (2)	0.0686 (13)	
H8A	0.1391	0.1679	1.1967	0.082*	
C9	−0.3390 (7)	0.4521 (4)	1.0869 (2)	0.0660 (12)	
H9A	−0.4173	0.5317	1.1033	0.079*	
H9B	−0.4490	0.3784	1.0877	0.079*	
C10	−0.2686 (7)	0.4773 (4)	1.0154 (2)	0.0558 (10)	
H10A	−0.4074	0.4910	0.9885	0.067*	
C11	−0.1210 (7)	0.3682 (4)	0.9839 (2)	0.0568 (10)	
H11A	−0.1071	0.2914	1.0134	0.068*	
H11B	−0.1892	0.3381	0.9427	0.068*	
C12	0.0875 (7)	0.5618 (4)	0.9838 (2)	0.0565 (10)	

### Atomic displacement parameters (Å^2^)

**Table:**

	*U*^11^	*U*^22^	*U*^33^	*U*^12^	*U*^13^	*U*^23^
N	0.0555 (19)	0.0374 (15)	0.084 (2)	0.0096 (16)	0.0052 (19)	−0.0044 (15)
O1	0.096 (2)	0.0555 (16)	0.0745 (18)	−0.0051 (18)	0.000 (2)	−0.0037 (14)
O2	0.0475 (15)	0.0404 (13)	0.121 (2)	0.0004 (13)	0.0102 (17)	−0.0137 (15)
O3	0.0402 (15)	0.0525 (16)	0.161 (3)	−0.0080 (15)	0.003 (2)	−0.0047 (18)
C1	0.081 (3)	0.082 (3)	0.077 (3)	0.000 (3)	0.000 (3)	−0.009 (3)
C2	0.079 (3)	0.063 (2)	0.076 (3)	−0.006 (3)	−0.001 (3)	−0.009 (2)
C3	0.067 (3)	0.058 (2)	0.054 (2)	−0.005 (2)	0.010 (2)	0.0022 (18)
C4	0.079 (3)	0.051 (2)	0.065 (2)	−0.007 (2)	0.002 (3)	−0.0078 (19)
C5	0.071 (3)	0.048 (2)	0.074 (3)	−0.001 (2)	0.011 (3)	−0.008 (2)
C6	0.066 (3)	0.053 (2)	0.059 (2)	−0.010 (2)	0.020 (2)	−0.0044 (18)
C7	0.083 (3)	0.048 (2)	0.064 (2)	−0.016 (2)	0.007 (3)	−0.0046 (18)
C8	0.100 (4)	0.045 (2)	0.061 (2)	−0.006 (3)	0.006 (3)	0.0016 (18)
C9	0.054 (2)	0.059 (2)	0.085 (3)	−0.003 (2)	0.015 (2)	−0.012 (2)
C10	0.0358 (18)	0.049 (2)	0.082 (3)	0.0002 (18)	−0.007 (2)	−0.005 (2)
C11	0.054 (2)	0.048 (2)	0.069 (2)	−0.010 (2)	−0.002 (2)	−0.0077 (18)
C12	0.044 (2)	0.0382 (18)	0.088 (3)	0.0028 (18)	−0.011 (2)	−0.0025 (19)

### Geometric parameters (Å, °)

**Table:**

N—C12	1.321 (5)	C4—C5	1.387 (6)
N—C10	1.444 (5)	C4—H4A	0.9300
N—H0A	0.8600	C5—C6	1.390 (6)
O1—C3	1.382 (5)	C5—H5A	0.9300
O1—C2	1.420 (5)	C6—C7	1.405 (6)
O2—C12	1.358 (5)	C6—C9	1.497 (6)
O2—C11	1.423 (5)	C7—C8	1.354 (7)
O3—C12	1.208 (5)	C7—H7A	0.9300
C1—C2	1.506 (6)	C8—H8A	0.9300
C1—H1A	0.9600	C9—C10	1.522 (6)
C1—H1B	0.9600	C9—H9A	0.9700
C1—H1C	0.9600	C9—H9B	0.9700
C2—H2A	0.9700	C10—C11	1.519 (5)
C2—H2B	0.9700	C10—H10A	0.9800
C3—C8	1.374 (6)	C11—H11A	0.9700
C3—C4	1.379 (6)	C11—H11B	0.9700
			
C12—N—C10	113.8 (3)	C7—C6—C9	123.1 (4)
C12—N—H0A	123.1	C8—C7—C6	122.7 (4)
C10—N—H0A	123.1	C8—C7—H7A	118.7
C3—O1—C2	117.1 (3)	C6—C7—H7A	118.7
C12—O2—C11	109.4 (3)	C7—C8—C3	120.6 (4)
C2—C1—H1A	109.5	C7—C8—H8A	119.7
C2—C1—H1B	109.5	C3—C8—H8A	119.7
H1A—C1—H1B	109.5	C6—C9—C10	115.1 (3)
C2—C1—H1C	109.5	C6—C9—H9A	108.5
H1A—C1—H1C	109.5	C10—C9—H9A	108.5
H1B—C1—H1C	109.5	C6—C9—H9B	108.5
O1—C2—C1	107.8 (4)	C10—C9—H9B	108.5
O1—C2—H2A	110.2	H9A—C9—H9B	107.5
C1—C2—H2A	110.2	N—C10—C11	100.2 (3)
O1—C2—H2B	110.2	N—C10—C9	113.0 (3)
C1—C2—H2B	110.2	C11—C10—C9	115.5 (3)
H2A—C2—H2B	108.5	N—C10—H10A	109.2
C8—C3—C4	119.5 (4)	C11—C10—H10A	109.2
C8—C3—O1	116.0 (4)	C9—C10—H10A	109.2
C4—C3—O1	124.4 (4)	O2—C11—C10	106.3 (3)
C3—C4—C5	119.1 (4)	O2—C11—H11A	110.5
C3—C4—H4A	120.5	C10—C11—H11A	110.5
C5—C4—H4A	120.5	O2—C11—H11B	110.5
C4—C5—C6	122.9 (4)	C10—C11—H11B	110.5
C4—C5—H5A	118.6	H11A—C11—H11B	108.7
C6—C5—H5A	118.6	O3—C12—N	129.3 (4)
C5—C6—C7	115.2 (4)	O3—C12—O2	121.3 (4)
C5—C6—C9	121.7 (4)	N—C12—O2	109.4 (4)
			
C3—O1—C2—C1	−178.3 (3)	C5—C6—C9—C10	85.5 (5)
C2—O1—C3—C8	−173.6 (4)	C7—C6—C9—C10	−92.8 (5)
C2—O1—C3—C4	6.2 (6)	C12—N—C10—C11	−5.5 (4)
C8—C3—C4—C5	0.3 (6)	C12—N—C10—C9	118.0 (4)
O1—C3—C4—C5	−179.5 (4)	C6—C9—C10—N	−62.6 (4)
C3—C4—C5—C6	−0.4 (6)	C6—C9—C10—C11	52.0 (5)
C4—C5—C6—C7	−0.3 (6)	C12—O2—C11—C10	−9.0 (5)
C4—C5—C6—C9	−178.8 (4)	N—C10—C11—O2	8.4 (4)
C5—C6—C7—C8	1.1 (6)	C9—C10—C11—O2	−113.4 (4)
C9—C6—C7—C8	179.5 (4)	C10—N—C12—O3	−179.6 (4)
C6—C7—C8—C3	−1.1 (7)	C10—N—C12—O2	0.3 (5)
C4—C3—C8—C7	0.4 (6)	C11—O2—C12—O3	−174.4 (4)
O1—C3—C8—C7	−179.7 (4)	C11—O2—C12—N	5.7 (5)

### Hydrogen-bond geometry (Å, °)

**Table:**

*D*—H···*A*	*D*—H	H···*A*	*D*···*A*	*D*—H···*A*
N—H0A···O3^i^	0.86	1.99	2.845 (4)	171
C10—H10A···O3^ii^	0.98	2.47	3.317 (5)	144

Symmetry codes: (i) *x*−1/2, −*y*+3/2, −*z*+2; (ii) *x*−1, *y*, *z*.
